# The association between daily steps and health, and the mediating role of body composition: a pedometer-based, cross-sectional study in an employed South African population

**DOI:** 10.1186/s12889-015-1381-6

**Published:** 2015-02-22

**Authors:** Julian D Pillay, Hidde P van der Ploeg, Tracy L Kolbe-Alexander, Karin I Proper, Maartje van Stralen, Simone A Tomaz, Willem van Mechelen, Estelle V Lambert

**Affiliations:** UCT/MRC Exercise Science and Sports Medicine Research Unit, Faculty of Health Sciences, University of Cape Town, Cape Town, South Africa; Department of Basic Medical Sciences, Faculty of Health Sciences, Durban University of Technology, Durban, South Africa; Department of Public and Occupational Health, EMGO+ Institute for Health and Care Research, VU University Medical Centre, Amsterdam, Netherlands

## Abstract

**Background:**

Walking is recognized as an easily accessible mode of physical activity and is therefore supported as a strategy to promote health and well-being. To complement walking, pedometers have been identified as a useful tool for monitoring ambulatory physical activity, typically measuring total steps/day. There is, however, little information concerning dose-response for health outcomes in relation to intensity or duration of sustained steps. We aimed to examine this relationship, along with factors that mediate it, among employed adults.

**Methods:**

A convenience sample, recruited from work-site health risk screening (N = 312, 37 ± 9 yrs), wore a pedometer for at least three consecutive days. Steps were classified as “aerobic” (≥100 steps/minute and ≥10 consecutive minutes) or “non-aerobic” (<100 steps/minute and/or <10 consecutive minutes). The data were sub-grouped according to intensity-based categories i.e. “no aerobic activity”, “low aerobic activity” (1-20 minutes/day of aerobic activity) and “high aerobic activity” (≥21 minutes/day of aerobic activity), with the latter used as a proxy for current PA guidelines (150-minutes of moderate-intensity PA per week). Health outcomes included blood pressure, body mass index, percentage body fat, waist circumference, blood cholesterol and blood glucose. Analysis of covariance, adjusting for age, gender and total steps/day were used to compare groups according to volume and intensity-based steps categories. A further analysis compared the mediation effect of body fat estimates (percentage body fat, body mass index and waist circumference) on the association between steps and health outcomes, independently.

**Results:**

Average steps/day were 6,574 ± 3,541; total steps/day were inversely associated with most health outcomes in the expected direction (p < 0.05). The “no aerobic activity” group was significantly different from the “low aerobic activity” and “high aerobic activity” in percentage body fat and diastolic blood pressure only (P < 0.05). Percentage body fat emerged as the strongest mediator of the relationship between steps and outcomes, while body mass index showed the least mediation effect.

**Conclusion:**

The study provides a presentation of cross-sectional pedometer data that relate to a combination of intensity and volume-based steps/day and its relationship to current guidelines. The integration of volume, intensity and duration of ambulatory physical activity in pedometer-based messages is of emerging relevance.

## Background

The association between physical inactivity and the increased risk of many clinical conditions has been well documented and is currently a major global public health concern [1,2]. Data from longitudinal cohort studies indicate at least a 1.5 to 2.0-fold higher risk of most chronic diseases of lifestyle with physical inactivity, such as coronary heart disease, type-2 diabetes, and hypertension [3-5].

The World Health Organization (WHO) physical activity guidelines recommend that individuals accumulate at least 150 minutes of moderate intensity physical activity (PA) per week (or equivalent), in bouts of at least 10 consecutive minutes in duration [6]. Such a recommendation is identified to result in a reduction in risk for all-cause mortality and disease-specific morbidity and mortality [6]. This dose-response effect encompasses components of PA including: mode, intensity, duration and frequency, and the expected response of improved health and well-being [7,8].

Walking has been reported as the most common mode of PA in both developed and developing countries [7,9-12]. This is, in part, due to the fact that walking is an inexpensive and easily accessible activity for a large portion of the general population [10] and across age groups [7]. Furthermore, there are fewer physical, social and psychological barriers associated with walking than with other forms of exercise [13].

Walking has been promoted, in part, by the growing popularity of pedometers and pedometer-linked health promotion messages, believed to be of Japanese origin and dating as far back as the 1960’s, that have suggested 10,000 steps per day (steps/day) as a target for health benefits [14-16].

A systematic review of 32 empirical studies suggests that relatively healthy adults take between 7,000-13,000 steps/day [17]. Tudor-Locke and Myers [18] have, however, suggested that 10,000 steps/day is unrealistically high for low-active or inactive adults and may therefore contribute to low program adherence. Furthermore, studies on the extent to which walking contributes to meeting PA guidelines, have generally presented volume-based steps/day information with limited information on the intensity of steps [19-21].

In the context of walking and steps/day recommendations, recent studies have been directed towards the application of intensity-based steps, such as a steps/minute rate for moderate intensity PA. For example, studies have shown that 30 minutes of moderate-to-vigorous walking equates to between 3,100 and 4,000 steps [22-24], even when considering factors such as stride length and body mass index in their recommendations [24,25]. Such studies have accordingly emphasized the significance of intensity-based steps/day recommendations as an emerging area of research. Further to this, recent studies make reference to intensity-based step recommendations and, in particular, an appropriate steps/minute rate for moderate intensity PA [23-25].

Information on ambulatory PA patterns, in the context of steps/day and, particularly, intensity-based steps which will add to the current understanding of the dose-response effects of walking. Such an application will support the use of intensity-based steps/day recommendations as an additional strategy/option for attaining current PA guidelines.

The aim of this study was, therefore, to determine the association between the volume and intensity of daily steps accumulated, and health measures in a South African employed adult group. The study also explores the extent to which body composition (percentage body fat, body mass index and waist circumference) may mediate the association between steps/day and clinical outcomes (blood pressure, blood cholesterol and blood glucose).

## Methods

The study was a cross-sectional study among South African employed adults.

### Participant recruitment

A convenience sample of participants was recruited through an invitation email sent out to employees, or following the completion of a health risk screening hosted at corporate organizations or private health facilities. The corporate organizations mainly comprised health insured, white-collared workers. The range of work performed by the employees varied considerably (from secretarial/administrative to senior management roles). The physical activity levels required by, and performed in most of these jobs were low in general. Upon completion of the informed consent form, participants were provided with a blinded pedometer.

### Pedometer wear

Participants were requested to wear an Omron HJ 720 ITC pedometer, attached to the left or right hip, as worn in most studies [26]. A 5-consecutive-day protocol was decided on as the number of days the participants would be requested to wear the pedometer. This would increase probability of obtaining at least three consecutive days of pedometer data, as a minimum criterion for estimating daily ambulatory PA [27-29].

The pedometer screen was covered to reduce the likelihood of participants observing their daily steps, which may have influenced habitual levels of PA and subsequently, daily steps accumulation during the study. Participants were asked to wear the pedometer throughout the day, to follow their usual routine of daily activities and to remove the pedometer only when bathing, showering or sleeping.

Participants were also informed that their daily results would be made available to them at the end of the study.

### Inclusion and exclusion criteria

Employees attending the health screening event and/or willing to participate in the study were eligible for inclusion. Other criteria included: being between the age of 21 years (inclusive) and 50 years (exclusive) and; willingness to wear a blinded pedometer, during waking hours, for the duration of the study.

Employees were excluded for the following reasons: pregnancy; diagnosis or treatment of cancer; any other condition that could impact PA; non-compliance to the pedometer wear and; participating in non-ambulatory PA (such as swimming and cycling) that may not be captured or may be inaccurate through the pedometer reading.

### Ethical considerations and informed consent

The study was approved by the Faculty of Health Sciences Research Ethics Committee (REC REF: 348/2008) of the University of Cape Town. Permission was also obtained from the corporate organizations to provide an on-site health screening event and invite employees to participate in the study. Employees were provided with a Participant Information Sheet, at the health screening event, detailing the purpose, aims, procedures, requirements and potential risks of the study. Thereafter, they were required to sign an Informed Consent Form.

### Body measures

Anthropometric measures were completed by a trained researcher/student as part of the health screening event.

Body height was measured in centimeters, using a height chart as the vertical distance from the floor to the vertex of the head. The participants stood barefoot with arms at their sides, and heels, buttocks and head in contact with the wall.

Waist circumference was measured (in centimeters) using a tape measure around the skin of the waist at the level of the umbilicus.

Body weight was measured using an electronic scale (Beurer® PS 06), allowing only a single layer of clothing. The values were rounded to the nearest 0.1 kg.

Body mass index was computed as weight (in kg) divided by height (in meters) squared.

The Omron Body Composition Monitor (BF500), which is based on the principles of bioelectrical impedance, was used to measure percentage body fat [30].

Blood pressure (BP) was recorded (in mmHg) using an electronic sphygmomanometer after the participant remained relaxed for five minutes. Two readings were taken, approximately five minutes apart. An average of the two readings was recorded. If the two readings obtained were different from each other (>5 mmHg), a third reading was taken. The average of the two nearest readings was used.

Finger prick blood cholesterol and blood glucose were taken using the Accutrend Plus® monitor device and measured in mmol/L.

### The Omron HJ 720 ITC pedometer

The Omron HJ 720 ITC (Omron Corp., Kyoto, Japan) is an example of a piezoelectric pedometer that provides information on intensity of steps taken, including a memory function that recalls previous data. The validity and reliability of this brand and model of pedometer has been studied at various mounting positions under prescribed and self-paced walking conditions with both healthy and overweight adults [31,32]. Such studies have promoted its application as an accurate measure of step counts [31,32].

A valuable feature of the pedometer is that the information captured by the pedometer can be uploaded electronically. The electronic display presents information as a visual display (bar graph) and further includes a 43 day recall with the ability to summarize information by weekly and monthly categories. A further benefit of this brand of pedometer is its ability to provide an hourly representation of steps data. The pedometer display differentiates hours that the pedometer was worn (even if no steps were accumulated) from hours that the pedometer was not worn.

Information provided on the pedometer display for immediate viewing, includes daily steps information, such as the total number of steps accumulated [33]. Additionally, the number of “manufacturer-defined aerobic steps” (>60 steps/minute, minimum duration of 1-minute), duration (in minutes) of “manufacturer-defined aerobic steps”, distance (in kilometers) completed and calories (kilocalories) expended are displayed [33]. The “manufacturer-defined aerobic steps” within the total steps/day record is therefore provided [33]. Consequently, total time spent accumulating such steps (in minutes per day (minutes/day)) is provided as aerobic time [33].

### Data recording

The pedometer data were uploaded electronically by the researcher according to the Omron Health Management Manager Software Protocol [33]. Feedback on pedometer data was communicated to the participant when the pedometer was being returned. Participants were included in the analyses if they had worn the pedometer for a minimum of three consecutive days and for at least ten hours on each of those days.

With particular reference to intensity-based steps, recently documented literature on intensity-based steps/day [23,34] suggests a minimum of 100 steps/minute to be a reliable estimate of, and target for, moderate intensity PA. Furthermore, the findings of recent pedometer-based studies [35,36] make reference to 100 steps/minute as a reliable indicator of moderate intensity PA.

Consequently, in our subsequent analysis, the 100 steps/minute rate for moderate intensity PA informed the refinement of “manufacturer-defined aerobic steps” to an estimate more relevant to current literature. Current literature also recommends that the accumulation of moderate intensity PA in bouts of at least ten minutes, is acceptable in contributing towards meeting current PA guidelines [6].

Thus, we limited our intensity-based analysis to bouts of ten consecutive minutes or more and at a minimum average intensity of 100 steps/minute, using the graphical display of pedometer results. “Manufacturer-defined aerobic steps” were used as a basis for separating sustained steps (≥60 steps/minute; duration ≥ 1 minute). This category of steps was further stratified according to whether these steps were sustained for at least ten minutes. We then determined the average intensity of these bouts (using the total number of steps and duration information). Steps accumulated at a minimum average intensity of 100 steps/minute were categorized as “aerobic steps”.

The data were further sub-grouped according to intensity-based categories i.e. “no aerobic activity”, “low aerobic activity” (1-20 minutes/day of aerobic activity) and “high aerobic activity” (≥21 minutes/day of aerobic activity). These sub-groups relate to current PA guidelines of 150 minutes of moderate intensity PA per week [6]. The guideline of accumulating a minimum of 150 minutes per week of moderate intensity PA [6-8] was translated to an average daily approximation of 21 minutes.

### Mediation analysis

The mediation effect of percentage body fat, body mass index and waist circumference on the association between both steps/day and aerobic time (as an estimate of moderate intensity PA) on clinical outcomes (systolic blood pressure, diastolic blood pressure, blood cholesterol and blood glucose) was examined.

**Figure 1:**
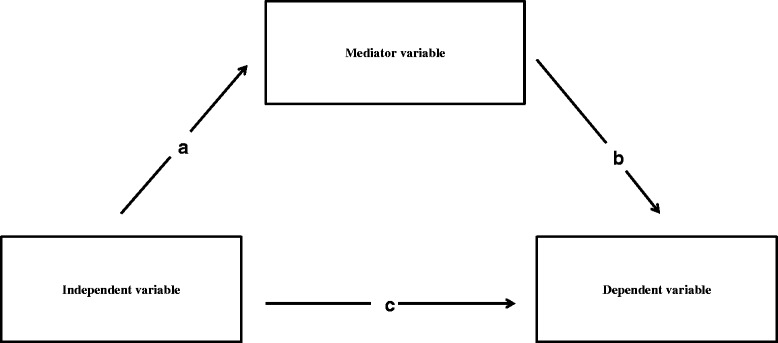
**Statistical mediation model.**

Our mediation analysis was informed by the product-of-coefficient test, which essentially consisted of four steps [37]. As a first step, we determined the total effect (c path) of the independent variable (e.g. steps/day) on the outcome variable (e.g. systolic blood pressure). We thereafter determined the association between the independent variable (e.g. steps/day) and the potential mediator (e.g. percentage body fat), calculated as the “a-coefficient”. Thirdly, the association between the potential mediator and the outcome variable was assessed (b-coefficient) and controlled for the independent variable (c’ coefficient). Figure 1 illustrates these steps.The c-prime (c’) path is the direct effect of the independent variable on the outcome variable, adjusted for the mediator. Finally, the mediated effects of the independent variable on the outcome variable through the proposed mediator, was determined by multiplying the a- and b coefficient (a*b coefficient). We also calculated the percentage mediation of the potential mediator (a*b/c) to assess the percentage of the effect of the independent variable on the outcome variable that could be explained by the mediator.

### Statistical analyses

The data were analyzed using STATISTICA version 8 (StatSoft Inc., Tulsa, OK, USA) and statistical significance was set at P < 0.05. General characteristics of the study group were summarized using descriptive statistics and additionally categorized by gender.

The data were subsequently grouped according to steps/day and aerobic time categories, respectively. An analysis of covariance (ANCOVA), adjusting for age, gender and total steps/day (when analyzing intensity-based steps) was used to compare groups based on volume and intensity-based steps categories. A post-hoc Bonferroni test was used to determine between group effects, where relevant.

For the mediation analysis, the bootstrapping statistical approach, using 1,000 bootstrap re-samples, was used, with the SPSS script of Preacher, Rucker and Hayes [38] to calculate the bias corrected confidence intervals (ab paths) around the mediated and direct associations [38].

## Results

### Participant characteristics

Of the 366 participants who volunteered to participate in the study, 312 participants (147 men and 165 women, 37 ± 9 yrs) wore the pedometer for a minimum of three consecutive days, for at least ten hours for each of those days, and were included in the analysis. Seventy-two participants had completed pedometer wear for three consecutive days, 109 for four consecutive days and 131 for five or more consecutive days. Eighty-nine participants had worn the pedometer for at least one weekend day and 40 participants for both weekend days.

The mean (±SD) steps/day accumulated in men and women was 7,476 ± 4,076 steps/day and 5,769 ± 2,759 steps/day, respectively.

Table 1 presents characteristics of the study group.

**Table 1:** **General Characteristics of participants**

**Variable**	**N**	**Total**	**n**	**Men**	**n**	**Women**
Age (years)	312	37.4 ± 9.3^*^	147	38.1 ± 9.9	165	36.8 ± 8.8
Height (m)	311	1.68 ± 0.1^**^	146	1.7 ± 0.1	165	1.6 ± 0.1
Weight (kg)	312	76.4 ± 16.8	144	81.1 ± 15.2	165	72.2 ± 17.1
Body mass index (kg/m^2^)	304	26.9 ± 5.7^*^	139	27.0 ± 4.9	165	26.8 ± 6.4
% body fat (%)	244	30.0 ± 10.7^**^	121	26.8 ± 8.8	123	33.2 ± 11.5
Waist circumference (cm)	302	85.8 ± 13.3	137	88.7 ± 12.1	165	83.4 ± 13.8
Systolic blood pressure (mmHg)	296	120.9 ± 14.5	131	125.5 ± 13.3	165	117.3 ± 14.4
Diastolic blood pressure (mmHg)	296	79.6 ± 11.7	131	81.1 ± 12.0	165	78.4 ± 11.3
Blood cholesterol (mmol/L)	250	3.9 ± 1.1	106	3.8 ± 0.9	144	4.0 ± 1.2
Blood glucose (mmol/L)	243	4.9 ± 1.8	100	4.9 ± 1.4	143	4.9 ± 2.1
Pedometer data (total steps/day)	312	6,574 ± 3,541^**^	147	7,476 ± 4,076	165	5,769 ± 2,759
Average daily aerobic steps (steps/day)	312	694 ± 1,465^**^	147	947 ± 1,771	165	468 ± 1,081
Average daily aerobic time (minutes/day)	312	5.7 ± 12.0^**^	147	7.8 ± 14.6	165	3.8 ± 8.7

Note: Values are means ± standard deviation.

*indicates statistical significance (P < 0.05), **(P < 0.01) between men and women.

The data were subsequently grouped according to four volume-based categories based on current steps/day classifications [27,34]: “<5,000 steps/day”; “5,000-7,499 steps/day”; “7,500-9,999 steps/day” and “≥10,000 steps/day” (Table 2).

**Table 2:** **Biometric and clinical measures by step per day categories**

**Variable**	**N**	**n**	<**5**,**000**	**n**	**5**,**000-** **7**,**499**	**n**	**7**,**500** **-9**,**999**	**n**	≥**10**,**000**
Weight (kg)	309	112	78.9 ± 19.0	99	75.4 ± 15.5	60	74.7 ± 17.2	38	73.8 ± 11.1
BMI (kg/m^2^)	304	110	28.1 ± 7.1	98	26.5 ± 5.1	59	25.7 ± 4.6	37	26.1 ± 3.6
% BF (%)	244	97	34.7 ± 12.5^B^	80	28.7 ± 8.3^A^	38	24.4 ± 7.2^A^	29	25.6 ± 7.7^A^
WC (cm)	302	111	89.9 ± 15.3^B^	99	84.8 ± 12.8^A^	56	81.3 ± 10.4^A^	36	83.0 ± 8.6^A^
SBP(mmHg)	296	111	122.2 ± 15.5	99	122.1 ± 15.0	55	117.9 ± 12.6	31	117.8 ± 11.1
DBP (mmHg)	296	111	81.9 ± 10.1	99	80.0 ± 13.1	55	76.1 ± 11.0	31	76.4 ± 11.5
BC (mmol/L)	250	99	4.1 ± 1.1	75	3.9 ± 1.2	47	3.7 ± 0.8	29	3.6 ± 0.8
BG(mmol/L)	243	98	5.4 ± 2.5^B^	71	4.5 ± 1.1^A^	45	4.4 ± 1.0^A^	29	4.8 ± 1.0^A^

Note: Values represent mean ± standard deviation, adjusted for age, gender and average daily aerobic steps.

Means with a different letter superscript indicate statistical significance between the groups compared (P < 0.05).

*BMI*- body mass index.

%BF- percentage body fat.

*WC*- waist circumference.

*SBP*- systolic blood pressure.

*DBP*- diastolic blood pressure.

*BC*- blood cholesterol.

*BG*- blood glucose.

A total of 112 participants (35.9%) accumulated an average of <5,000 steps/day, typically classified as inactive [27,34]. Only 38 participants (12.2%) achieved an average of ≥10,000 steps/day, typically classified as “active” [27,34]. Within the “active” group, most of the participants (73%) also accumulated aerobic steps.

As illustrated in Table 2, the <5,000 steps/day group was significantly different from all other groups (P < 0.05) in percentage body fat, waist circumference and blood glucose only, when adjusted for age, gender and daily aerobic steps (so as to establish the independent effect of volume of steps/day).

Table 3 presents our data according to intensity-based categories, i.e. “no aerobic activity”; “low aerobic activity” and “high aerobic activity”.

**Table 3:** **Biometric and clinical measures based on daily aerobic step time categories**

**Variable**	**N**	**n**	**No aerobic time**	**n**	**1** **-21 minutes/day**	**n**	≥**21 minutes/day**
Weight (kg)	309	209	76.7 ± 17.5	66	76.8 ± 15.4	34	73.6 ± 15.2
BMI (kg/m^2^)	304	207	27.1 ± 6.1	63	26.9 ± 5.1	34	25.6 ± 4.6
% BF (%)	244	168	32.2 ± 11.6^B^	49	26.2 ± 6.6^A^	27	23.7 ± 5.7^A^
WC (cm)	302	207	86.9 ± 14.0	63	84.0 ± 11.9	32	82.5 ± 10.6
SBP(mmHg)	296	204	121.8 ± 15.0	60	119.4 ± 12.7	32	118.4 ± 13.9
DBP (mmHg)	296	204	80.9 ± 11.4^B^	60	77.4 ± 10.9^A^	32	75.2 ± 13.3^A^
BC (mmol/L)	250	183	4.0 ± 1.1	42	3.8 ± 0.9	25	3.7 ± 0.8
BG(mmol/L)	243	180	5.0 ± 2.1	39	4.5 ± 0.9	24	4.7 ± 1.8

Note: Values represent mean ± standard deviation, adjusted for age and gender and total steps/day.

Means with a different letter superscript indicate statistical significance between groups (P < 0.05).

*BMI*- body mass index.

%*BF*- percentage body fat.

*WC*- waist circumference.

*SBP*- systolic blood pressure.

*DBP*- diastolic blood pressure.

*BC*- blood cholesterol.

*BG*- blood glucose.

Of the total sample, 102 participants (32.7%) accumulated aerobic steps. Only 34 participants (11.0%) of the total study group, however, accumulated aerobic steps for a minimum duration of 21 minutes/day.

When adjusted for age, gender and total steps/day (to establish the independent effect of intensity-based steps), the “no aerobic activity” group was significantly different from the “low aerobic activity” and the “high aerobic activity” in percentage body fat and diastolic blood pressure only (P > 0.05). Table 3 illustrates these differences.

A subsequent analysis investigated the mediation effect of variables related to body composition, i.e. percentage body fat, body mass index and waist circumference, on the association between volume and intensity-based steps/day and clinical outcomes (systolic blood pressure, diastolic blood pressure, blood cholesterol and blood glucose).

Table 4 presents a summary of the results.

**Table 4:** **Mediation effect of percentage body fat**, **body mass index and waist circumference on the association between total daily steps** **/aerobic time and clinical measures**

**Mediator**	**Independent variable**	**Outcome Variable**	**c path** **(SE)**	**a path** **(SE)**	**b path** **(SE)**	**c’** **path** **(SE)**	**ab path** **(95%CI)**	**% mediation**
Percentage body fat	Total steps (steps/day)							
		SBP (mmHg)(n = 233)	**-2.70** **(0.94)**	**-3.61** **(0.67)**	**0.33** **(0.94)**	-1.50(0.98)	**-1.19** **(-2.46;-0.53)**	44
		DBP(mmHg)(n = 233)	**-2.75** **(0.78)**	**-3.61** **(0.67)**	**0.28** **(0.08)**	**-1.76** **(0.81)**	**-0.99** **(-1.63;-0.49)**	36
		BC (mmol/L)(n = 195)	**-0.18** **(0.08)**	**-3.44** **(0.74)**	**0.02** **(0.01)**	-0.11(0.08)	**-0.07** **(-0.16;-0.0006)**	38
		BG (mmol/L)(n = 189)	**-0.31** **(0.14)**	**-3.37** **(0.74)**	**0.04** **(0.01)**	-0.19(0.15)	**-0.13** **(-0.35;-0.24)**	42
	Aerobic time (minutes/day)							
		SBP (mmHg)(n = 233)	**-3.71** **(1.37)**	**-4.39** **(0.98)**	**0.34** **(0.09)**	-2.22(1.39)	**-1.48** **(-2.72;-0.70)**	60
		DBP (mmHg)(n = 233)	**-4.34** **(1.12v)**	**-4.39** **(0.98)**	**0.27** **(0.07)**	**-3.14** **(1.14)**	**-1.19** **(-1.98;-0.67)**	27
		BC (mmol/L)(n = 195)	-0.18(0.13)	**-4.00** **(1.17)**	**0.02** **(0.01)**	-0.09(0.13)	**-0.08** **(-0.19;-0.01)**	44
		BG (mmol/L)(n = 189)	-0.30(0.23)	**-4.13** **(1.20)**	**0.04** **(0.01)**	-0.13(0.23)	**0.17** **(-0.46;-0.04)**	57
Body mass index	Total steps (steps/day)							
		SBP (mmHg)(n = 290)	**-2.38** **(0.84)**	**-1.04** **(0.34)**	**0.60** **(0.14)**	-1.75(0.82)	**-0.63** **(-1.31;-0.20)**	26
		DBP(mmHg)(n = 290)	**-2.61** **(0.69)**	-**1.04** **(0.34)**	**0.51** **(0.11)**	**-2.08** **(0.68)**	**-0.53** **(-0.95;-0.20)**	20
		BC (mmol/L)(n = 247)	**-0.16** **(0.07)**	-0.71(0.38)	0.01(0.01)	**-0.15** **(0.07)**	-0.009(-0.05;0.005)	06
		BG (mmol/L)(n = 240)	**-0.27** **(0.12)**	**-0.77** **(0.38)**	**0.07** **(0.02)**	**-0.27** **(0.12)**	**-0.05** **(-0.17;-0.007)**	19
	Aerobic time (minutes/day)							
		SBP (mmHg)(n = 290)	**-2.81** **(1.17)**	-0.67(0.50)	**0.63** **(0.14)**	**-2.38** **(1.17)**	-0.43(-1.18;0.10)	15
		DBP (mmHg)(n = 290)	**-3.40** **(0.10)**	-0.67(0.50)	**0.54** **(0.11)**	**-3.03** **(0.96)**	-0.36(-0.87;0.09)	11
		BC (mmol/L)(n = 247)	-0.12(0.11)	-0.38(0.57)	0.02(0.01)	-0.12(0.11)	-0.01(-0.05;0.01)	08
		BG (mmol/L)(n = 240)	-0.25(-0.18)	-0.37(0.60)	**0.07** **(0.02)**	-0.22(0.18)	-0.03(-0.14;0.04)	12
Mediator	Independent variable	Outcome Variable	c path (SE)	a path (SE)	b path (SE)	c’ path (SE)	ab path (95% CI)	% mediation
Waist circumference	Total steps (steps/day)							
		SBP (mmHg)(n = 292)	**-2.60** **(0.83)**	**-3.91** **(0.76)**	**0.34** **(0.06)**	-1.26(0.82)	**-1.34** **(-2.21;-0.76)**	52
		DBP(mmHg)(n = 292)	**-2.90** **(0.67)**	**-3.91** **(0.76)**	**0.27** **(0.05)**	**-1.83** **(0.67)**	**-1.05** **(-1.65;-0.60)**	36
		BC (mmol/L)(n = 243)	**-0.15** **(0.07)**	**-3.43** **(0.83)**	0.01(0.01)	-0.12(0.07)	-0.03(-0.09;0.001)	20
		BG (mmol/L)(n = 236)	**-0.30** **(0.12)**	**-3.34** **(0.84)**	**0.03** **(0.01)**	-0.20(0.12)	**-0.10** **(-0.19;-0.05)**	33
	Aerobic time (minutes/day)							
		SBP (mmHg)(n = 292)	**-3.30** **(1.21)**	**-3.24** **(1.14)**	**0.35** **(0.06)**	-2.15(1.16)	**-1.14** **(-2.07;-0.46)**	35
		DBP (mmHg)(n = 292)	**-3.91** **(0.99)**	**-3.24** **(1.14)**	**0.28** **(0.05)**	**-2.99** **(0.95)**	**-0.91** **(-1.62;-0.36)**	23
		BC (mmol/L)(n = 243)	-0.12(0.11)	**-3.01** **(1.31)**	**0.01** **(0.01)**	-0.01(0.11)	**-0.03** **(-0.10;-0.0002)**	25
		BG (mmol/L)(n = 236)	-0.25(0.19)	**-3.31** **(1.33)**	**0.03** **(0.01)**	-0.14(0.19)	**-0.11** **(-0.24;-0.03)**	44

c = total effect of the independent variables (i.e. steps/day and aerobic time) on the outcome variable (i.e SBP, DBP, BC, BG),

a = association between the independent variable (e.g. steps/day) and the potential mediator (e.g. percentage body fat).

b = association between the potential mediator and the outcome variable was assessed, controlled for the independent variable.

c’ = direct effect of the independent variable on the outcome variable was determined.

ab = indirect effects of the independent variable on the outcome variable through the proposed mediator.

% mediation = percentage mediation of the potential mediator (a*b/c).

*SE*: Standard error.

values represented in bold indicate significant associations.

SBP- systolic blood pressure.

DBP- diastolic blood pressure.

BC- blood cholesterol.

BG- blood glucose.

As indicated by the c path (direct effect), our results clearly show that body composition mediates the association between PA and health outcomes. However, as indicated by the c’ path, the direct effect of nearly all the outcome variables when adjusted for the mediator, was no longer significant.

Percentage body fat was the strongest mediator (27-60% mediation, in associations of statistical significance) in the association between steps (steps/day and aerobic activity) and clinical measures. Waist circumference also mediated most of the associations between steps and clinical measures, although providing a weaker extent of mediation (23-52% mediation, in associations of statistical significance) than percentage body fat. Body mass index was evidently a less convincing mediator in the associations presented (19-26% mediation, in association where statistical significance was found).

## Discussion

The mean (±SD) steps/day of 6,574 ± 3,541 suggests that our sample group fell below the lower end of 7,000-13,000 steps/day for healthy, younger adults [17]. Our data were, nevertheless, consistent with the previous observation that individuals accumulating <5,000 steps/day are more likely to be classified as obese [39]. Significant differences were also found between some biometric (percentage body fat, waist circumference) and clinical (blood glucose) outcomes in those participants in the <5,000 steps/day category, when compared with the other steps/day categories. As recently confirmed [14], total daily values less than 5,000 steps/day may be an appropriate index for inactivity and its associated risk with health consequences, such as obesity.

Additional findings were that 102 participants (32.7%) accumulated aerobic steps (≥100 steps/min for a minimum duration of 10-minutes/day). Only 34 participants (11.0%), however, accumulated aerobic steps for an average of at least 21 minutes/day, as a proxy for current PA guidelines that make reference to moderate intensity PA. This suggests that only approximately one-third of our study group accumulated some moderate intensity PA relevant to the current PA in respect to ambulatory PA [6].

The most notable finding was that most participants accumulating more than 10,000 steps/day were also accumulating “aerobic” steps. A direct and independent association of intensity-based steps was, however, only found in percentage body fat and diastolic blood pressure. The contribution of intensity-based steps towards achieving 10,000 steps/day however, highlights the value of intensity-based steps as a contributor towards achieving volume-based recommendations. Our research, therefore, emphasizes and supports emerging literature that exercise prescription and/or steps/day recommendations be framed within the context of volume, intensity and duration of intensity-based steps rather than volume alone [35,36].

Recent studies have shown that 30 minutes of moderate-to-vigorous walking corresponds to a total of between 3,000 and 4,000 steps [22,27,34,40]. In support of our additional findings, reference to 3,000 steps in 30 minutes was, therefore made as an overall guideline that incorporates volume, duration and intensity of steps/day. The use of 3,000 steps in 30 minutes is, however suggested as a heuristic value and taken over and above habitual activity levels. This recommendation therefore still supports the accumulation of volume-based steps and the 10,000 steps/day recommendation.

Whilst our study group may not be truly representative of the South African adult population, the results do support the viewpoint that globally, most adults are currently not meeting PA guidelines [41].

### Association between steps per day and body composition

Research has shown that people meeting the 10,000 steps/day target are more frequently classified as normal weight and those individuals with values less than 5,000 steps/day are more frequently classified as obese [39]. In addition, a distinct relationship between steps/day and body composition variables in the expected direction [39,42-47] has been previously reported.

In general, we did not find significant differences in body composition between participants accumulating more than 10,000 steps/day versus those accumulating 7,500-9,999 steps/day or even 5,000-7,499 steps/day. There was, however, significance between the <5,000 steps/day group and the other three groups in percentage body fat and waist circumference (adjusted for age, gender and aerobic steps, so as to establish the true effect of volume of steps/day). Similarly, there was a significant difference between the “no aerobic time” group and the other two groups in percentage body fat (adjusted for age, gender and total steps/day, so as to establish the independent effect of intensity based steps), suggesting that “some” intensity-based steps is better than “none”.

### Steps per day in relation to moderate intensity physical activity and current physical activity guidelines

The categorization of our pedometer data into intensity-based steps categories described in the methodology so as to relate to current PA guidelines, showed conflicting findings. After adjusting for age, gender and total steps/day (to establish the independent effect of intensity-based steps), only percentage body fat and diastolic blood pressure were significantly different between the “no aerobic activity” and the “low aerobic activity” groups. A similar finding was observed when comparisons between the “no aerobic activity” and the “high aerobic activity” groups were made. No other between-group effects were noted. This may direct us to the notion that “some physical activity is better than none” [45].

Furthermore, the observation that most people accumulating 10,000 steps also accumulated intensity-based steps directs us to the viewpoint that intensity-based steps contributes to improved outcomes by increasing total volume of steps/day.

Tudor-Locke et al, in a recent paper [14], identifies the gap in current literature on the impact of intensity-based walking programs and on clinical outcomes, within the context of pedometry. Whilst pedometer-based walking programs have shown increased walking behavior and varying levels of improvement in clinical outcomes, research on the impact of intensity-based walking programs and their effect on clinical outcomes, is of emerging importance. Our study, in exploring the interplay between volume and intensity-based steps/day, supports this recommendation.

### Mediation effect of waist circumference, percentage body fat and body mass index in the association between steps per day and clinical outcomes

Mediation analysis has emerged as a statistical technique for providing insights into the mechanisms of change, particularly in behavioral interventions. As such, in the area of PA and health, mediation analysis has been used to determine which behaviors contribute to weight loss. The notion that regular physical activity is associated with lower body fat composition (body mass index, percentage body fat and waist circumference) and improved clinical measures is well documented. Resting Energy Expenditure and the variation thereof is, however, largely due to differences in the extent of lean body mass and fat mass of an individual [48]. Consequently, all associations between body size and other outcomes (such as cardiovascular risk factors) are partially confounded by the association with resting energy expenditure [48]. Increased levels of PA are seen to increase energy expenditure and improve clinical measures, such as reducing blood pressure [48].

After accounting for PA, the positive correlation between increased resting energy expenditure and blood pressure, for example [49] may direct us to the viewpoint that the association between PA and clinical outcomes might be mediated by factors relating to body fat.

From our mediation analysis, it is evident that the relationship between steps/day (both total steps/day and aerobic time) and clinical outcomes (blood pressure, blood cholesterol and blood glucose) were influenced (mediated) by body composition estimates. In our study, percentage body fat emerged as the strongest and significant mediator in this association, and may, therefore be a useful consideration in the associations between PA measures and clinical outcomes, particularly as a criterion measure for body composition.

Whilst our results have clearly shown that body composition mediates the association between PA and health outcomes, the loss of significance of nearly all the outcome variables when adjusted for the mediator, further highlights that body composition (and most notably, percentage body fat) “completely”, rather than “partially”, mediates the association between PA and health outcomes.

Although such a finding has valuable and far-reaching implications on PA and health, the information presented may not, however, be able to draw inferences to causality, due to the cross-sectional nature of the data.

### Strengths of the study

The research undertaken is, to our knowledge, among the first pedometer-based studies conducted in the Republic of South Africa, within an urban context, that establishes the association between ambulatory PA and health measures in an adult, employed population group.

The study was useful in establishing associations between volume, intensity (in terms of aerobic steps/day accumulated) and duration (total time spent in aerobic activity) of ambulatory PA and health measures.

A number of studies that have directly measured moderate intensity as 3 METs have concluded that 100 steps/minute is a reasonable heuristic value, indicative of moderate intensity PA [14,34,50]. The application of the 100 steps/minute criterion within our data analyses provides a unique presentation of cross-sectional pedometer data that relates to a combination of intensity and volume-based steps/day rather than volume alone.

The study, by way of the mediation analysis, provides an interesting finding on the role of physical activity in improving health through improved body composition.

### Limitations

The cross-sectional nature of the study provided information on the association between ambulatory PA and clinical outcomes. No causal inference could therefore be drawn.

The study was limited to those employees attending the health screening event and/or willing to participate in the study. This presents a selection bias, as the group agreeing to participate may be different from the non-participating employees. We were also not able to obtain data on the response rate of those invited to participate, and those that were excluded in the analysis.

Pedometers measure ambulatory PA. Our study was, therefore, limited to participants performing activities more specific to ambulation and did not include activities such as swimming, cycling and weight training.

The low steps/day volume noted in our study (6,574 + 3,541) may be related to the paucity of the data obtained (i.e. three consecutive days of pedometer-wear, minimum wear-time of ten hours). The wearing time is in keeping with documented literature that make reference to such criteria as a reasonable estimate of daily ambulatory PA [51,28,29].

The categorization of manufacturer-defined “aerobic” steps, from the hourly display into a more representative estimate of moderate intensity PA (i.e. 100 steps/minute in bouts of at least ten minutes), can be seen as a limitation due to the manual method by which this re-categorization of the data was performed.

Consequently, the sub-grouping of the data according to intensity-based categories using 21 minutes/day of aerobic activity, as a proxy for current PA guidelines, may be viewed as a further limitation, as this sub-grouping is similarly based on the manual tallying of data. This criterion has, however, allowed us to provide some level of differentiation of PA according to total volume of steps/day and aerobic time.

## Conclusions

The findings of this research highlight the effect of volume and intensity of steps/day on health outcomes. The mediation effect of typical measures of body composition on the association between daily steps and health outcomes is also demonstrated. This mediation effect highlights the importance of PA in improving health by improving body composition.

The study adds to current documented literature in providing some information on intensity-based steps/day in a cross-sectional study. Our study additionally provides a unique application of steps/minute recommendations to our data. In so doing, our findings relate closely to current PA guidelines by adopting an appropriate estimate of moderate intensity PA, as part of our criterion in the data analyses. The consequent integration of volume, intensity and duration of ambulatory PA in pedometer-based guidelines, is of emerging relevance as an option for meeting current guidelines.

The mediation analysis, in highlighting that body composition completely mediates the association between physical activity and health outcomes, provides an interesting and unique observation that may have far-reaching implications on PA and health.

Further studies using a similar but broader approach can be adopted so as to provide prevalence data rather than data through convenience sampling.

## Abbreviations

ANCOVA: Analysis of covariance
BC: Blood cholesterol
BG: Blood glucose
BMI: Body mass index
BP: Blood pressure
DBP: Diastolic blood pressure
minutes/day: minutes per day
%: percentage
PA: Physical activity
%BF: Percentage body fat
steps/day: Steps per day
steps/minute: Steps per minute
SBP: Systolic blood pressure
WC: Waist circumference
WHO: World Health Organization

## References

[1] Alberti KG, Zimmet P, Shaw J (2005). The metabolic syndrome-a new worldwide definition. Lancet.

[2] Wang F, McDonald T, Champagne LJ, Edington DW (2004). Relationship of body mass index and physical activ¬ity to health care costs among employees. J Occup Environ Med.

[3] Lambert EV, da Silva R, Fatti L, Patel D, Kolbe-Alexander T, Derman W (2009). Fitness-related activities and medical claims related to hospital admissions -South Africa, 2006. Prev Chronic Dis.

[4] Rodgers A, Ezzati M, Van der Hoorn S, Lopez AD, Lin RB, Murray CJ (2004). Distribution of major health risks: findings from the Global Burden of Disease Study. PLoS Med.

[5] Brown WJ, Burton NW, Rowan PJ (2007). Updating the evidence on physical activity and health in women. Am J Prev Med.

[6] WHO. Global recommendations on physical activity for health. 2010. http://whqlibdoc.who.int/publications/2010/9789241599979_eng.pdf. Accessed 11 July 2015 26180873

[7] Haskell WL, I-Min L, Pate RR, Powell KE, Blair SN, Franklin BA (2007). Physical Activity and Public Health: updated recommendation for adults from the American College of Sports Medicine and the American Heart Association. Med Sc Sports Exerc.

[8] Chief Medical Office, Department of Health, London (2004). At least five a week: evidence on the impact of physical activity and its relationship to health- a report from the Chief Medical Officer.

[9] Tudor-Locke CE, Myers AM (2001). Methodological considerations for researchers and practitioners using pedometers to measure physical (ambulatory) activity. Res Q Exerc Sport.

[10] Hallal PC, Azevedo MR, Reichert FF, Siqueira FV, Araujo CL, Victora CG (2005). Who, when, and how much? Epidemiology of walking in a middle-income country. Am. J. Prev. Med.

[11] Blair SN (1984). How to assess exercise habits and physical fitness. Behavioral health: a handbook of health enhancement and disease prevention.

[12] Masse LC, Ainsworth BE, Tortolero S, Levin S, Fulton JE, Karla A (1998). Measuring physical activity in midlife, older and minority women. Womens Health.

[13] Alexander S, Cowburn G, Foster C (2006). Understanding participation in sport and physical activity among children and adults: a review of qualitative studies. Health Educ Res.

[14] Tudor-Locke C, Craig CL, Brown WJ, Clemes SA, De Cocker K, Gile-Corti B (2011). How many steps/day are enough? For adults. Int J Behav Nutr Phys Act.

[15] Hatano Y, Tudor-Locke C (2001). Pedometer-assessed physical activity: measurement and motivations.

[16] Hatano Y (1993). Use of the pedometer for promoting daily walking exercise. Int Council Health Phys. Educ. Recreation.

[17] Bravata DM, Smith-Spangler CS, Sundaram V, Gienger AL, Lin N, Lewis R (2007). Using pedometers to increase physical activity and improve health- a systematic review. JAMA.

[18] Tudor-Locke CE, Meyers AM (2001). Challenges and opportunities for measuring physical activity in sedentary adults. Sports Med.

[19] Merom D, Phongsaven P, Chey T, Bauman A (2006). Long-term changes in leisure time walking, moderate and vigorous exercise: were they influences by national physical activity guideline?. Med Sci Sports Exerc.

[20] Bergman P, Grjibovsci AM, Hagstromer M, Bauman A, Sjostrom M (2008). Adherence to physical activity recommendations and the influence of socio-demographic correlates- a population-based cross-sectional study. BMC Public Health.

[21] Schmidt MD, Cleland VJ, Shaw K, Dwyer T, Venn AJ (2009). Cardiometabolic risk in younger and older adults across an index of ambulatory activity. Am J Prev Med.

[22] Wilde BE, Sidman CL, Corbin CB (2001). A 10,000 step count as a physical activity target for sedentary women. Res Q Exerc Sport.

[23] Marshall SJ, Levy SS, Tudor-Locke CE, Kolkhorst FW, Wooten KM, Ji M (2009). Translating physical activity recommendations into a pedometer-based step goal: 3000 steps in 30 minutes. Am J Prev Med.

[24] Rowe DA, Welk GJ, Heil DP, Mahar MT, Kemble CD, Calabro MA (2011). Stride rate recommendations for moderate intensity walking. Med Sci Sports Exerc.

[25] Beets MW, Agiovlasitis S, Fahs CA, Ranadive SM, Fernhall B (2010). Adjusting step count recommendations for anthropometric variations in leg length. J Sc Med Sport.

[26] Tudor-Locke C (2002). Taking steps toward increased physical activity: using pedometers to measure and motivate.

[27] Tudor-Locke C, Bassett DRJ (2004). How many steps/d are enough? Preliminary pedometer indices for public health. Sports Med.

[28] Rowe DA, Kemble CD, Robinson TS, Mahar MT (2007). Daily walking in older adults: day-to-day variability and criterion-referenced validity of total daily step counts. J Phys Act Health.

[29] Hart TL, Swartz AM, Cashin SE, Strath SJ (2011). How many days of monitoring predict physical activity and sedentary behaviour in older adults?. Int J Behav Nutr Phys Act.

[30] Bosy-Westphal A, Later W, Hitze B, Sato T, Kossel E, Gluer CC (2008). Accuracy of bioelectrical impedence consumer devices for measurement of body composition in comparison to whole body magnetic resonance imaging and dual x-ray absorptiometry. Obes Facts.

[31] Hasson RE, Haller J, Pober DM, Staudenmayer J (2009). Validity of the Omron HJ-112 pedometer during treadmill walking. Med Sci Sports Exerc.

[32] Holbrook EA, Barreira TV, Kang M (2009). Validity and reliability of Omron pedometers for prescribed and self-paced walking. Med Sci Sports Exerc.

[33] Incorporated Omron Health Management. Instruction manual: Pocket pedometer-model HJ-720ITC, 2007

[34] Tudor-Locke C, Sisson SB, Collova T, Lee SM, Swan PD (2005). Pedometer-determined step count guidelines for classifying walking intensity in a young ostensibly healthy population. Can J Appl Physiol.

[35] Pillay JD, Kolbe-Alexander TL, Proper KI, van Mechelen W, Lambert EV (2014). Steps that count- Physical activity recommendations, brisk walking and steps per minute- how do they relate?. J Phys Act Health.

[36] Pillay JD, Kolbe-Alexander TL, van Mechelen W, Lambert EV (2014). Steps that count- the association between the number and intensity of steps accumulated and fitness and health measures. J Phys Act Health.

[37] MacKinnon DP, ed *Introduction to statistical mediation analysis*. Mahway, NJ,2008.

[38] Preacher KJ, Rucker DD, Hayes AF (2007). Addressing moderated mediation hypotheses: theory, methods, and prescriptions. Multivar Behav Res.

[39] Tudor-Locke C, Ainsworth BE, Whitt MC, Thompson RW, Addy CL, Jones DA (2001). Relationship between pedometer-determined ambulatory and body composition variables. Int J Obes.

[40] Welk GJ, Differding JA, Thompson RW, Blair SN, Dziura J, Hart P (2000). The utility of the Digi-walker step counter to assess daily physical activity patterns. Med Sci Sports Exerc.

[41] Hallal PC, Andersen LB, Bull FC, Guthold R, Haskell W, Ekelund U (2012). Global physical activity levels: surveillance progress, pitfalls and prospects. Lancet.

[42] McClung CD, Zahiri CA, Higa JK, Amstutz HC, Schmalzried TP (2000). Relationship between body mass index and activity in hip or arthoplastypatients. J Orthop Res.

[43] Rowlands AV, Eston RG, Ingledew DK (1994). Relationship between activity levels, aerobic fitness, and body fat in 8-10 year old children. J Appl Physiol.

[44] Chan CB, Spangler E, Valcour J, Tudor-Locke C (2003). Cross-sectional relationship of pedometer-determined ambulatory activity to indicators of health. Obes Res.

[45] US Department of Helath Services. Physical Activity Guidelines for Americans: Be Active, Healthy, and Happy! Washington, D.C,2008.

[46] Le Masurier GC, Sidman CL, Corbin CB (2003). Accumulating 10,000 steps: does this meet current physical activity guidelines?. Res Q.

[47] Harber V, Bell G, Rodgers W, Courneya KS (2006). Cardiovascular and Type 2 diabetes risk factor response to traditional fitness and 10,000 step exercise program: the health 1st study. Med Sci Sports Exerc.

[48] Nelson KM, Weinsier RL, Long CL, Schutz Y (1992). Prediction of resting energy expenditure from fat-free mass and fat mass. Am J Clin Nutr.

[49] Luke A, Adeyemo A, Kramer H, Forrester T, Cooper RS (2004). Association between blood pressure and resting energy expenditure independent of body size. Hypertension.

[50] Abel M, Hannon J, Mullineaux D, Beighle A (2011). Determination of step rate thresholds corresponding to physical activity intensity classifications in adults. J Phys Act Health.

[51] Kang M, Marshall SJ, Barreira TV, Lee J (2009). Effect of pedometer-based physical activity interventions: a meta-analysis. Res Q.

